# The spatial and temporal fidelity in real-time MRI in patients with sinus rhythm and arrhythmias

**DOI:** 10.1186/1532-429X-16-S1-O11

**Published:** 2014-01-16

**Authors:** Francisco Contijoch, Kelly Rogers, Walter R Witschey, Robert C Gorman, Yuchi Han

**Affiliations:** 1 University of Pennyslvania, Philadelphia, Pennsylvania, USA

## Background

Irregularly irregular arrhythmia is difficult to image with conventional cine CMR because arrhythmia rejection and registration-based reconstruction result in cardiac motion artifacts or unachievable breath-hold durations. Undersampled radial trajectories, combined with a suitable non-Cartesian reconstruction algorithm, have the potential to overcome these problems by sampling a small number of radial projections without loss of temporal fidelity associated with temporal regularization. Despite the large number of available real-time reconstruction algorithms and sampling trajectories, there are very few reports regarding optimal values for basic reconstruction parameters such as image exposure time (radial projections per image frame) and temporal resolution (view sharing). Our goal was to address the problem of spatial and temporal fidelity in real-time MRI using automatic active contour segmentation in subjects with sinus rhythm and also patients with irregularly irregular arrhythmias.

## Methods

Short-axis stacks of images were acquired in 20 subjects using standard retrospective gated bSSFP cine imaging and a golden-angle radial bSSFP trajectory (total views = 4000, views per image = 34) reconstructed into 128 × 128 images via conjugate gradient-SENSE based iterative reconstruction (Gadgetron). Golden-angle radial acquisitions can be reconstructed with a variable number of projections (quantified as the exposure time Te of each frame). In addition, projections can be shared between two adjacent frames resulting in higher reconstructed temporal resolution. Two datasets were reconstructed with varying exposure time (Te = 28 - 840 ms) and temporal resolution (Tr = 2.8 - 96.2 ms) to evaluate the effect on ejection fraction (EF). Global function was assessed via semi-automated segmentation of all phases and all left ventricular slices (ITK-SNAP, Philadelphia PA) in the short-axis stack and EDV, ESV, SV, and EF were measured.

## Results

**Figure 1:**
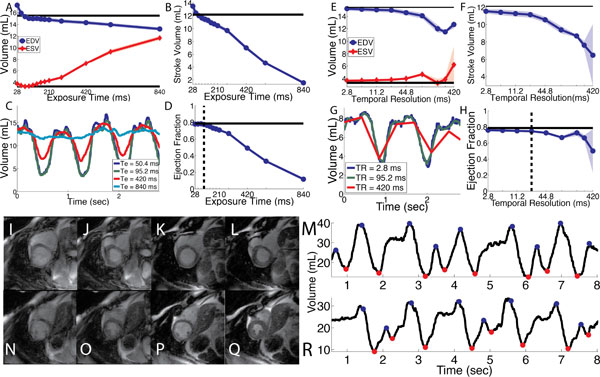
**Panel A-D illustrate the effect of exposure time on measure volumetric values**. Increasing the exposure time improves k-space sampling but results in temporal blurring. Panels E-H illustrate the effect of reconstructed frame rate on measure values. As the frame rate decreases, the local maxima and minima values are not obtained leading to changes in hemodynamic evaluation. The proposed method was utilized to image patients with frequent irregular arrhythmias. The improvement in image quality is shown for two different slice locations Panel I-L and N-Q. Standard EKG-gated images are shown at end-diastole (I and N) and end-systole (J and O). The real-time method shows improved image quality at both phases (end-diastole: K and P, end systole: L and Q). Furthermore, by obtaining volume for 8 seconds, the occurrence of premature beats can be observed (M and R). End-diastolic points are labeled blue and end-systolic points are labeled with a red dot.

EF is sensitive to both exposure time and temporal resolution and we found an Te < 100 ms and Tr < 22.8 ms is necessary to avoid substantial error. The figure illustrates how errors accumulate beyond these values. With these values (Te = 95.2 ms, Tr = 2.8 ms), we did not observe significant differences in EF (real time = 46.7 ± 20.1%, cine = 47.2 ± 21.9% p = 0.6282,) or stroke volume (real time = 62.0 ± 17.8%, cine = 64.3 ± 23.9%, p = 0.4746) in 11 subjects with sinus rhythm. In 9 subjects with rhythm disturbances, volume curves (illustrated in the Figure 1) were obtained which show the effect of arrhythmic beats. EF quantified in these patients, the estimated EF was higher than that from low quality CINE images.

## Conclusions

Accurate volume assessment at high spatiotemporal fidelity is possible in patients with irregular arrhythmias with real-time CMR and active contour segmentation. Our estimated values for optimal image exposure time and temporal resolution will guide future consensus regarding these basic reconstruction parameters.

## Funding

K99-HL108157, R01-HL103723, T32HL007954, T32-EB009384.

